# The role of point-of-care 3-hydroxybutyrate testing in patients with type 2 diabetes undergoing coronary angiography

**DOI:** 10.1007/s40618-017-0615-0

**Published:** 2017-02-11

**Authors:** S. Vigili de Kreutzenberg, A. Avogaro

**Affiliations:** Department of Medicine, University of Padova, Via Giustiniani, 2, 35128 Padua, Italy

**Keywords:** Ketone Bodies, Fasting, 3-Hydroxybutyrate, Coronary heart disease, Point-of-care

## Abstract

**Purpose:**

Ketone bodies, 3-hydroxybutyrate (3BOHB), and acetoacetate derive from increased free fatty acid beta-oxidation, thus reflecting marked insulin deprivation with or without decompensated diabetes. Objectives of this study were (1) to determine circulating levels of 3BOHB in patients with and without type 2 diabetes (T2DM), before and after an elective coronary angiography; (2) to detect 3BOHB modification during the procedure; (3) to study possible associations between 3BOHB and clinical parameters/outcomes.

**Methods:**

Sixteen T2DM (72 ± 11 years) and 22 matched controls (71 ± 12 years) undergoing elective coronary angiography were enrolled. In all subjects, biohumoral parameters were determined at hospital admission. Point-of-care determinations of 3BOHB, glucose, and creatinine were performed, at 7 a.m, immediately before and after the procedure. The duration of the fasting period and of the procedure was recorded.

**Results:**

T2DM had significantly higher fasting (0.538 ± 0.320 vs 0.255 ± 0.197 mM/l;   *p* = 0.005) and pre-procedural (0.725 ± 0.429 vs 0.314 ± 0.205; *p* = 0.002) 3BOHB concentrations than controls. Similarly, absolute increment of 3BOHB from the morning value was significantly greater in T2DM (0.369 ± 0.252 vs 0.127 ± 0.135 in controls; *p* = 0.002). Significant correlations were observed between pre-procedure 3BOHB and glucose levels (*r* = 0.586; *p* < 0.0001) and between pre-procedure 3BOHB and fasting creatinine concentrations (*r* = 0.364; *p* = 0.029).

**Conclusions:**

An overnight fasting period and a concomitantly stressful condition induce inappropriate 3BOHB increase in T2DM. Point-of-care capillary 3BOHB may be useful before any procedural/surgical intervention in these patients.

## Introduction

Ketone bodies (KB), acetoacetate (AcAc) and 3-hydroxybutyrate (3BOHB), are metabolites produced by free fatty acid (FFA) beta-oxidation, and utilized by peripheral cells as an energy source [1, 2]. Ketogenesis is stimulated when circulating FFA is increased from lipolysis, such as during either prolonged fasting or adrenergic activation [3].

Although blood KB concentrations in healthy individuals are low, they can increase 100 folds during diabetic ketoacidosis [4]. Recently, there has been a resurgent interest about these substrates, since ketoacidosis may be a rare complication of sodium-glucose co-transporter 2 (SGLT2) inhibitors [5]; moreover, it was shown that 3BOHB is an endogenous inhibitor of histone deacetylases (HDACs) and its metabolites, acetyl-CoA and succinyl-CoA, have themselves signalling activities [6].

Several years ago Lommi and colleagues reported increased concentration of KB in chronic heart failure (CHF), in proportion to the severity of cardiac dysfunction and neurohormonal activation [7]. In a subsequent study, the same authors suggested that severe CHF is a ketosis-prone state determined by augmented supply of FFA, induced by increased stress hormone-related lipolysis [8]: after 20 h of fasting, the reported total KB concentrations in patients with CHF were 600 µmol/l as compared to 350 µmol/l in controls. On the other hand, KB production can also represent an efficient energy substrate for the heart, providing more cellular energy per unit of oxygen consumed than glucose or FFA [9]. Myocardial KB metabolism maintains cardiac protein acetylation and ROS homeostasis, in the injured heart [10]. However, the normal heart may counter-regulate against ‘excessive’ metabolism of ketone bodies in the presence of increased production [11].

Our hypothesis is that type 2 diabetic patients undergoing coronary angiography/revascularization procedures for coronary heart disease (CHD) may show increased KB levels, depending on stress, diabetes control, and fasting duration.

Therefore, the objectives of this pilot study were (1) to determine the concentrations of capillary 3BOHB in patients with suspected CHD, with and without type 2 diabetes, undergoing elective coronary angiography, either with or without revascularization; (2) to establish the effect of fasting duration on 3BOHB concentrations; and (3) to detect 3BOHB modification during the procedure.

## Subjects and methods

### Subjects

In this prospective, cohort study, 16 type 2 diabetic patients (T2DM) and 22 matched controls admitted to Hospital, eligible for elective coronary angiography, were enrolled. Diabetes status was defined as (1) ongoing antidiabetic treatment; and (2) a HbA1c > .5% (48 mmol/mol). The decision for PTCA during coronary angiography was left to the discretion of interventional cardiologist. Recruitment started on January 2013, and ended on September 2015. Elective angiography was deemed necessary to rule out CHD, as for clinical indication. Patients with factors that could influence the lipid profile (alcohol abuse, thyroid disease, kidney, and liver disease) were excluded from the study. All patients received aspirin (100 mg daily) for the entire study period. Exclusion criteria were type 1 diabetes, active immunologic, neoplastic, and major organ diseases/failure.

Patients were maintained on their usual therapy, and consumed a weight maintaining diet containing at least 200 grams of carbohydrates per day for at least 2 days before the study. All patients enrolled in the study were on anti-platelets agents, statin therapy, renin-angiotensin blockers.

Informed consent was obtained from each subject. The study protocol was approved by the local Institutional Review Board.

### Study protocol

At hospital admission, in each patient, the following parameters were collected: sex, age, height, weight, BMI, arterial blood pressure, heart rate, family history of CHD and diabetes, the presence of cardiovascular disease (CVD) or arterial hypertension, smoke habit, and active therapy. A fasting blood sample for the determination of plasma glucose, glycated haemoglobin, creatinine, CKD-EPI estimated glomerular filtration rate (eGFR), total cholesterol, HDL cholesterol, triglyceride, troponin I, and BNP was collected. LDL cholesterol was calculated by Friedwald formula; urine was collected for the determination of microalbuminuria.

Determinations of 3BOHB, glucose (StatStrip® XpressMeter Gluc/Ket—Nova Biomedical), and creatinine (StatSensor Xpress® CREAT—Nova Biomedical) were performed on venous blood, at 7 a.m, the morning of the coronary angiography, and then immediately before (3BOHB and glucose only) and after the procedure. The duration of the fasting period and of the procedure was recorded.

Elective coronary angiography was performed using the standard technique by percutaneous femoral or radial approach. All patients were pretreated with aspirin and clopidogrel. At the beginning of the procedure, a bolus of unfractionated heparin was administered in order to achieve an activated clotting time of >250 s; heparin was stopped at the end of the procedure in the absence of coexisting indications such as bedside treatment with an intra-aortic balloon pump. Cardiac injury markers were routinely measured.

### Analytical determinations

The determination of blood glucose, 3BHOB, and creatinine was performed with a point-of-care approach, applying a drop of venous blood to the StatStrip® XpressMeter Gluc/Ket and StatSensor Xpress® CREAT—Nova Biomedical, as per manufacturer’s instructions for immediate testing. The StatStrip® XpressMeter Gluc/Ket and StatSensor Xpress® CREAT (http://www.novabiomedical.com/products/statsensor-creatinine/) measurements were performed in duplicate for glucose, 3BOHB, and creatinine. Testing methodological aspects are reported elsewhere [12, 13]. In this study, we measured whole blood beta hydroxybutyrate levels as this is the principal and most abundant ketone body found in ketoacidosis [14] and has previously been shown to be a reliable indicator of altered mitochondrial redox state and ketone body production during fasting [7, 15–17]. The performance of whole blood tests including StatStrip Ketone has previously been validated against laboratory methods in published studies [12, 18, 19]. In these studies, StatStrip Ketone has demonstrated a good correlation to the reference laboratory methods used and also to an GCMS definitive traceable method used for measuring for low levels of BOHB in metabolic conditions [20]. Based on these studies, it was considered that StatStrip Ketone was acceptable for use in the study without the need for further analytical evaluation.

Plasma glucose was determined by the glucose oxidase method; creatinine by the Jaffe method. Glycated haemoglobin was determined with HPLC procedure; total cholesterol, HDL cholesterol, and triglyceride were measured by enzymatic colorimetric test. BNP and troponin I were determined by immunoenzymatic methods. Microalbuminuria was dosed by a turbidimetric method.

### Statistical analysis

Study Size. A total of 38 patients (16 with diabetes and 22 without diabetes) were recruited in this study, allowing a probability of 80% that the study detected a blood 3BOHB concentration difference at a two-sided 0.05 significance level, between diabetic and non-diabetic subjects, if the true difference between groups is 50 µmol/l. This is based on the assumption that the standard deviation of the response variable is 0.5. Data are expressed as mean ± standard deviation. Normal distribution was verified with the Kolmogorov–Smirnov test. Differences between the two groups were assessed using the Student* t* test for unpaired data or Mann–Whitney test for non-normally distributed variables. Univariate correlations were checked using the Pearson’s *r* coefficient. Data are expressed as mean ± SD. Statistical significance was accepted at *p* < 0.05 and the SPSS version 21.0 (IBM SPSS statistics for Windows, Version 21.0 Armonk, NY, IBM Corp.) was used.

## Results

Table 1 summarizes demographic, clinical, and biochemical parameters of the study subjects.

**Table 1:** Main demographic, clinical, and biochemical parameters of the study subjects

Variable	Type 2 diabetes (*n* = 16)	Controls (*n* = 22)	*p*
Age (years)	72 ± 11	71 ± 12	0.673
Female (%)	25	32	0.847
BMI (kg/m^2^)	28.0 ± 3.4	26.4 ± 2.9	0.124
Blood Pressure (systolic/diastolic) (mm Hg)	141 ± 16/79 ± 9	147 ± 17/82 ± 8	0.218/0.325
Heart rate (bpm)	83 ± 15	81 ± 14	0.790
History of hypertension (%)	94	77	0.049
Stable angina (%)	13	5	0.359
Heart failure^a^ (%)	33	27	0.692
Previous revascularization (%)	13	14	0.918
Troponin I (µg/l)	0.38 ± 0.86	0.11 ± 0.24	0.168
BNP (pg/ml)	534 ± 679	348 ± 552	0.367
Duration of diabetes (years)	12 ± 5	NA	–
eGFR (ml/min/1.73 m^2^) (CKD-EPI)	69 ± 25	88 ± 33	0.063
Microalbuminuria (mg/24h)	123 ± 105	32 ± 46	0.004
HbA1c (%) (mmol/mol)	7.8 ± 3.9 (62 ± 20)	5.7 ± 0.2 (39 ± 2)	0.026
Total cholesterol (mg/dl)	163 ± 44	176 ± 44	0.370
HDL cholesterol (mg/dl)	42 ± 12	47 ± 9	0.087
LDL cholesterol (mg/dl)	107 ± 40	121 ± 40	0.296
Triglycerides (mg/dl)	138 ± 94	111 ± 37	0.288
Active Smoking (%)	19	45	0.0867
Insulin therapy (%)	27	–	–
Oral antidiabetic agents^b^ (%)	69	–	–
Incretins^c^ (%)	27	–	–
CHD demonstrated by CAG (*n*) (%)	(87)	(50)	0.0161

*BMI* body mass index, *HDL* high-density lipoprotein, *LDL* low-density lipoprotein, *eGFR* estimated glomerular filtration rate, *CHD* coronary heart disease, *CAG* coronary angiography

^a^Heart failure was defined by a BNP value >300 pg/ml

^b^Either metformin, SUs, Glitazones

^c^Either dipeptidyl peptidase 4 inhibitors or glucagon like peptide-1 receptor agonist

Type 2 diabetic patients were comparable to controls in demographical indexes. However, they had significantly lower eGFR and significantly higher microalbuminuria excretion rate. No differences were observed in troponin I and BNP circulating levels.

As expected, diabetic patients had significantly higher fasting plasma glucose than controls.

The length of fasting to procedure was almost identical between the two groups (T2DM 849 ± 163 vs 787 ± 29 min; *p* = 0.085); however, the length of procedure was significantly longer in diabetic patients (76 ± 36 min) than in controls (46 ± 23 min; *p* = 0.007).

**Fig. 1:**
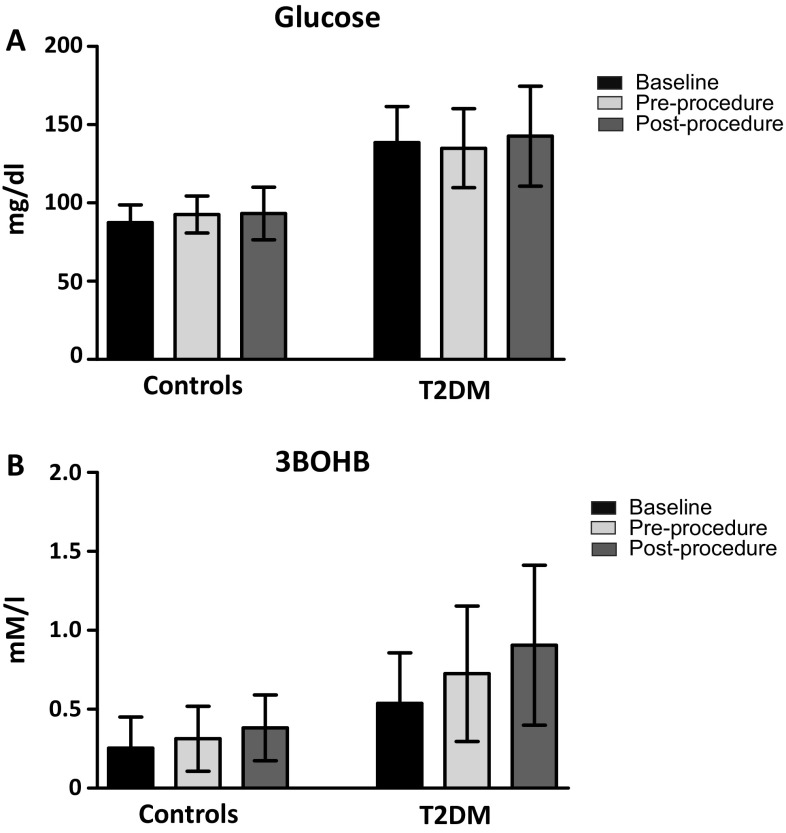
Plasma glucose (**a**) and 3BOHB (**b**) levels in controls and type 2 diabetic patients (T2DM), in three different occasions: at baseline, immediately pre-procedure and immediately post-procedure. *All *p* < 0.001 vs controls

Figure 1 illustrates glucose and 3BOHB concentrations, in the three different conditions (i.e. baseline, pre-, and post-procedure) in T2DM patients and in control subjects. As indicated in Fig. 1b, diabetic patients had significantly higher fasting 3BOHB concentration than controls (0.538 ± 0.320 vs 0.255 ± 0.197 mM/l; *p* = 0.005). Also pre-procedural 3BOHB concentration was significantly higher in diabetic patients than controls (0.725 ± 0.429 vs 0.314 ± 0.205 mM/l; *p* = 0.002). Post-procedural 3BOHB was also significantly higher in the former group, with an absolute increment of 3BOHB from the morning value of 0.369 ± 0.252 (0.127 ± 0.135 nM/l in controls; *p* = 0.002). This increment is due to an enhanced ketone body production in diabetes, rather than to a reduced clearance, as already demonstrated [21]. However, there was no correlation between 3BOHB increment and procedure duration.

**Fig. 2:**
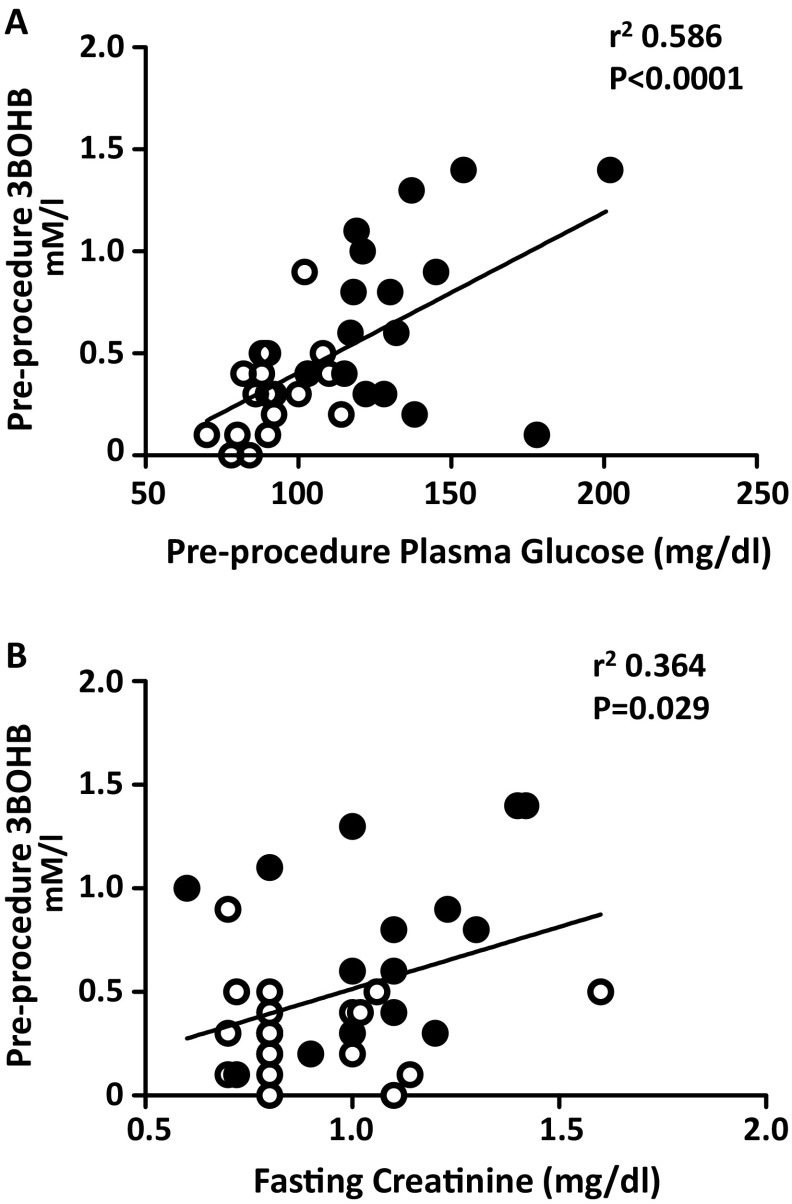
Correlations between pre-procedure glucose and pre-procedure 3BOHB (**a**), and between fasting creatinine and pre-procedure 3BOHB (**b**). (*White dots* controls; *black dots* T2DM subjects)

After pooling the results of the two groups, significant correlations were observed between pre-procedural glucose and pre-procedural 3BOHB (*r* ^2^ = 0.586; *p* < 0.0001) (Fig. 2a), and between fasting plasma glucose and post-procedural 3BOHB concentrations (*r* ^2^ = 0.554; *p* < 0.0001). Significant correlations were also observed between fasting creatinine and pre-procedural 3BOHB levels (*r* ^2^ = 0.364; *p* = 0.029) (Fig. 2b), between post-procedural creatinine and post-procedural 3BOHB concentrations (*r* ^2^ = 0.423; *p* = 0.010), and between pre-procedural 3BHOB concentration and creatinine increment during the procedure (*r* ^2^ = 0.512; *p* = 0.001).

A positive correlation was observed between fasting duration and fasting plasma glucose levels (*r* ^2^ = 0.392; *p* < 0.015). On the other hand, we were unable to demonstrate an association between fasting duration and 3BOHB levels, probably due to the small range of fasting length.

Coronary angiography (CAG) revealed a coronary heart disease (CHD) in 25 subjects (66% of the whole cohort), of which 16% had one vessel involvement, 16% two vessels, and 34% three vessels. Twelve patients (32%) underwent a PTCA, and 10 patients (26%) a CABG. Comparing T2DM and controls, the former showed a significantly higher prevalence of CHD (*n* 14; 87% vs *n* 11; 50%; *p* = 0.0161) (Table 1) and of three-vessel disease (56 vs 18%; *p* = 0.0146). We then compared metabolic parameters in patients with vs without CHD. Comparing subjects with vs without CHD, no differences were observed for sex, age, blood pressure values, glycated haemoglobin, lipid profile, troponin, BNP, while eGFR (72 ± 26 vs 95 ± 34 ml/min/1.73 m^2^; *p* = 0.032) and microalbuminuria (88 ± 99 vs 37 ± 47 mg/24h; *p* = 0.038) were significantly different in CHD subjects. Analysing glucose, 3BOHB, and creatinine values before and after CAG, pre-procedure 3BOB, and creatinine were significantly higher in patients with CHD, in the entire cohort (Table 2); post-procedure creatinine and its increase during the procedure were also higher in CHD subjects, while 3BOH increase and post-procedure values did not reach the statistical significance, although more elevated in the CHD group (Table 2). No significant variations were observed for glucose values. Fasting and procedure duration was similar in CHD and non-CHD patients (Table 2).

**Table 2:** Comparisons of metabolic parameters between subjects with CHD vs without CHD, as documented by coronary angiography

Parameter	Without CHD (*n* 25)	With CHD (*n* 13)	*p*
Pre-procedure glucose (mg/dl)	102 ± 26	115 ± 29	0.191
Post-procedure glucose (mg/dl)	100 ± 21	121 ± 38	0.076
Pre-procedure 3BOHB (mM/l)	0.35 ± 0.21	0.56 ± 0.42	0.044
Post-procedure 3BOHB (mM/l)	0.46 ± 0.25	0.68 ± 0.51	0.088
Pre-procedure creatinine (mg/dl)	0.85 ± 0.14	1.02 ± 0.26	0.015
Post-procedure creatinine (mg/dl)	1.00 ± 0.25	1.30 ± 0.33	0.009
Δglucose (mg/dl)	−2 ± 19	6 ± 11	0.105
Δ3BOHB (mM/l)	0.16 ± 0.20	0.26 ± 0.23	0.187
Δcreatinine (mg/dl)	0.15 ± 0.17	0.28 ± 0.18	0.050
Fasting duration (min)	816 ± 117	812 ± 110	0.918
Procedure duration (min)	47 ± 27	64 ± 34	0.127

## Discussion

The main findings of this study are that (1) blood 3BOHB concentrations are significantly higher in T2DM patients than in controls; (2) 3BOHB levels increase more sharply in the former group during a stress condition, such as an invasive diagnostic procedure, i.e. coronary angiography planned to either confirm or exclude CHD. The higher 3BOHB concentration was observed despite a comparable fasting period before the angiographic procedure. Even higher concentrations were observed after the procedure, although the length of this was significantly higher in diabetic patients. These results, obtained in a relatively small cohort of subjects, further stress the robustness of the observation, highlighting the metabolic role of these substrates also in T2DM, and the need of their determination, in a simple and fast manner, such as a point-of-care approach, in particular settings, to guarantee an optimal clinical management of diabetes.

We used the point-of-care determination of 3BOHB, which has been shown to be equally sensitive in detecting diabetic ketoacidosis in humans [22–24]; the capillary 3BOHB determination is easy and rapid to perform and gives objective results, which may improve management of the diabetic patient, especially in upsetting conditions [25]. Thus, the point-of-care capillary 3BOHB determination may be useful before any procedural/surgical intervention in T2DM patients.

Our data not only indicate that patients with type 2 diabetes may show increased KB concentration, but expand the findings of Lommi and colleagues, who showed that CHF had elevated blood KB (median 267 µmol/l) compared with control subjects (median 150 µmol/l) [7]. We were unable to find any correlation between BNP, which identified patients with cardiac-related dyspnoea with a sensitivity of 99% and a specificity of 41% [26], and 3BOHB concentration; however, our findings support an increased circulating 3BOHB concentration in T2DM patients independently of the presence of overt heart failure [15]. Notably, pooling T2DM and non-diabetic subjects, those with CHD, documented at CAG, showed higher basal levels of both 3BOB and creatinine, but not of glucose: to corroborate this finding, a significant correlation was observed between fasting troponin and 3BOHB values (*r* ^2^ 0.454; *p* = 0.005). Increased levels of 3BOHB have been described in both patients with and without diabetes with ischemic heart disease [27], as well as heart failure: it is well known that ketone bodies represent an alternative fuel source in the failing heart.

The present data also support our previous findings showing that blood levels of ketones are increased in T2DM patients, in spite of higher plasma insulin and no differences in plasma levels of cortisol, and growth hormone [28]. Fasting and stressful conditions further stimulate KB metabolism in diabetic patients, and excessive ketogenesis can lead to critical ketoacidosis; therefore, the availability of a simple point-of-care KB determination in peculiar clinical conditions is advised to monitoring the metabolic status of the patient. The analytical approach used herein detects only 3BOHB and not acetoacetate: however, in conditions where KB are increased, there is a progressive shift of acetoacetate to 3BOHB so that the latter ketone is by far the most representative [29].

As shown in Fig. 2a, we show a direct, linear correlation between pre-procedural 3BOHB concentration and plasma glucose: this finding has important clinical implications since it underscores the need of a strict glycaemic control when a diagnostic procedure such as coronary angiography is planned in patients with diabetes. In this context, KB were found to be increased during general anaesthesia without surgical stress as a consequence of prolonged fasting [30]. More recently, increased KB concentrations were observed in children younger than 36 months, who can present with ketoacidosis and (low) normal blood glucose concentrations before surgical intervention: these authors emphasize, at least in this group of patients, a better optimization of fasting time, in accordance with current guidelines [31].

Another finding, which deserves attention, is the direct correlation between creatinine levels and 3BHOB concentration both in fasting condition (Fig. 2b), and post-procedure, and also between 3BHOB concentration and creatinine increment during the procedure. Regrettably, there is no available report explaining the effect of chronic kidney disease on KB levels, and further studies are needed to corroborate this observation. On the contrary, it has been found that an acute increase in blood concentration of KB increases glomerular filtration rate in both control and in patients with diabetes patients, but it may cause a tubular proteinuria [32]. Recently, it was found that enhanced KB production in the diabetic kidney may represent a mechanism involved in the pathogenesis of diabetic nephropathy [33].

The increased blood 3BOHB concentrations in our patients with diabetes may be determined by increased adrenergic activation secondary either to the stressful condition, and/or to the fasting condition. Beside the well-known effect of catecholamines on lipolysis, and the secondary rise in KB production, it has been shown that stressor situations may increase KB by more than 400% [34]: this increase, observed in normal weight subjects, was associated with a surge in ACTH, norepinephrine and epinephrine concentrations, which unfortunately we were unable to assess. Also fasting is a well-known condition characterized by a progressive increase in circulating KB concentration [35]; the co-existence of both stress and prolonged fasting, probably in combination with the state of insulin resistance of T2DM patients, leads to higher 3BOHB levels in this group of subjects.

This study, albeit small, has however important clinical implications, the first being that the fasting state before an invasive and stressful procedure is characterized by an inappropriate elevation of circulating KB level. Although well below a life-threatening threshold, this increase in 3BOHB should be considered since, if not recognized properly, it may predispose to a more serious increase in its concentration [36]. These results also should suggest reducing the length of the fasting period before any procedural or surgical intervention in hospitalized T2DM patients; additionally, a diet richer in carbohydrates should be implemented in these patients, especially in those with SGLT2 inhibitors, which can potentially increase 3BOHB reabsorption [5]. Notably, it has been recently hypothesized that in mild, persistent hyperketonemia, such as those that prevail during treatment with SGLT2 inhibitors, 3BOHB is freely taken up by the heart and oxidized in preference to fatty acids, being, this, a positive fuel selection to improve the transduction of oxygen consumption into work efficiency at the mitochondrial level [37]. However, this theory has been criticized on biochemical ground [38]. It must be acknowledged that one of the limitation of this study is that only 3BOHB was determined, and this may not reflect the whole concentration of KB; additionally, the pre-procedural fasting duration could not be sufficient long to determine a significant increase of 3BOHB concentration.

In conclusion, a point-of-care and easy detection of 3BOHB may represent an additional and relatively simple assay, which may assist the physician to avoid metabolic decompensation in hospitalized T2DM patients before and after invasive and stressful procedures.

## References

[1] Alberti KG, Johnston DG, Gill A, Barnes AJ, Orskov H (1978). Hormonal regulation of ketone-body metabolism in man. Biochem Soc Symp.

[2] Laffel L (1999). Ketone bodies: a review of physiology, pathophysiology and application of monitoring to diabetes. Diabetes Metab Res Rev.

[3] Williamson DH (1981). Mechanisms for the regulation of ketogenesis. Proc Nutr Soc.

[4] Kitabchi AE, Wall BM (1995). Diabetic ketoacidosis. Med Clin North Am.

[5] Peters AL, Buschur EO, Buse JB, Cohan P, Diner JC, Hirsch IB (2015). Euglycemic diabetic ketoacidosis: a potential complication of treatment with sodium-glucose cotransporter 2 inhibition. Diabetes Care.

[6] Newman JC, Verdin E (2014). beta-hydroxybutyrate: much more than a metabolite. Diabetes Res Clin Pract.

[7] Lommi J, Kupari M, Koskinen P (1996). Blood ketone bodies in congestive heart failure. J Am Coll Cardiol.

[8] Lommi J, Koskinen P, Näveri H, Härkönen M, Kupari M (1997). Heart failure ketosis. J Intern Med.

[9] Cahill JF, Veech RL (2003). Ketoacids? Good medicine?. Trans Am Clin Climatol Assoc.

[10] Schugar RC, Moll AR, d’Avignon DA (2014). Cardiomyocyte-specific deficiency of ketone body metabolism promotes accelerated pathological remodeling. Mol Metab.

[11] Wentz AE, d’Avignon DA, Weber ML (2010). Adaptation of myocardial substrate metabolism to a ketogenic nutrient environment. J Biol Chem.

[12] Ceriotti F, Kaczmarek E, Guerra E (2015). Comparative performance assessment of point-of-care testing devices for measuring glucose and ketones at the patient bedside. J Diabetes Sci Technol.

[13] Minnings K, Kerns E, Fiore M (2015). Chronic kidney disease prevalence in Rivas, Nicaragua: Use of a field device for creatinine measurement. Clin Biochem.

[14] Gosmanov AR, Gosmanova EO, Dillard-Cannon E (2014). Management of adult diabetic ketoacidosis. Diabetes Metab Syndr Obes.

[15] Avogaro A, Valerio A, Gnudi L (1992). Ketone body metabolism in NIDDM. Effect of sulfonylurea treatment. Diabetes.

[16] Snorek M, Hodyc D, Sedivý V (2012). Short-term fasting reduces the extent of myocardial infarction and incidence of reperfusion arrhythmias in rats. Physiol Res.

[17] Service FJ, O’Brien PC (2005). Increasing serum betahydroxybutyrate concentrations during the 72-hour fast: evidence against hyperinsulinemic hypoglycemia. J Clin Endocrinol Metab.

[18] Aitkenhead H, Marwaha K, Evans S (2010). Assessment of the accuracy and performance of handheld POC sensors for measuring whole blood 3-hydroxybutyrate. Point Care.

[19] Arkir Z, Webber K, Davis K (2013). Evaluation of POC methods for measurement of ketones in metabolic medicine. Point Care.

[20] Schulz S, Tendl K, Bohn A (2014). Point of care whole blood ketone measurement traceability to an GCMS reference measurement procedure. Clin Chem Lab Med.

[21] Balasse EO, Féry F (1989). Ketone body production and disposal: effects of fasting, diabetes, and exercise. Diabetes Metab Rev.

[22] Taboulet P, Haas L, Porcher R (2004). Urinary acetoacetate or capillary beta-hydroxybutyrate for the diagnosis of ketoacidosis in the Emergency Department setting. Eur J Emerg Med.

[23] Arora S, Henderson SO, Long T, Menchine M (2011). Diagnostic accuracy of point-of-care testing for diabetic ketoacidosis at emergency-department triage: {beta}-hydroxybutyrate versus the urine dipstick. Diabetes Care.

[24] Klocker AA, Phelan H, Twigg SM, Craig ME (2013). Blood beta-hydroxybutyrate vs. urine acetoacetate testing for the prevention and management of ketoacidosis in Type 1 diabetes: a systematic review. Diabet Med.

[25] Guerci B, Tubiana-Rufi N, Bauduceau B (2005). Advantages to using capillary blood beta-hydroxybutyrate determination for the detection and treatment of diabetic ketosis. Diabetes Metab.

[26] Chenevier-Gobeaux C, Guerin S, André S (2010). Midregional pro-atrial natriuretic peptide for the diagnosis of cardiac-related dyspnea according to renal function in the emergency department: a comparison with B-type natriuretic peptide (BNP) and N-terminal proBNP. Clin Chem.

[27] Lopaschuk GD, Ussher JR, Folmes CD (2010). Myocardial fatty acid metabolism in health and disease. Physiol Rev.

[28] Avogaro A, Crepaldi C, Miola M (1996). High blood ketone body concentration in type 2 non-insulin dependent diabetic patients. J Endocrinol Invest.

[29] Avogaro A, Doria A, Gnudi L (1992). Forearm ketone body metabolism in normal and in insulin-dependent diabetic patients. Am J Physiol.

[30] Tarhan S, Fulton RE, Moffitt EA (1971). Body metabolism during general anesthesia without superimposed surgical stress. Anesth Analg.

[31] Dennhardt N, Beck C, Huber D (2015). Impact of preoperative fasting times on blood glucose concentration, ketone bodies and acid-base balance in children younger than 36 months: a prospective observational study. Eur J Anaesthesiol.

[32] Trevisan R, Nosadini R, Fioretto P (1987). Ketone bodies increase glomerular filtration rate in normal man and in patients with type 1 (insulin-dependent) diabetes mellitus. Diabetologia.

[33] Zhang J, Yang H, Kong X (2011). Proteomics analysis reveals diabetic kidney as a ketogenic organ in type 2 diabetes. Am J Physiol Endocrinol Metab.

[34] Kubera B, Hubold C, Wischnath Z, Zug S, Peters A (2014). Rise of ketone bodies with psychosocial stress in normal weight men. Psychoneuroendocrinology.

[35] Akram M (2013). A focused review of the role of ketone bodies in health and disease. J Med Food.

[36] Wilson JF (2010) In clinic. Diabetic ketoacidosis. Ann Intern Med 152:ITC1-1- ITC1-15, table of contents; quiz ITC1-16. 10.7326/0003-4819-152-1-201001050-01001 20048266

[37] Ferrannini E, Mark M, Mayoux E (2016). CV protection in the EMPA-REG OUTCOME trial: a “thrifty substrate hypothesis”. Diabetes Care.

[38] Lopaschuk GD, Verma S (2016). Empagliflozin’s fuel hypothesis: not so soon. Cell Metab.

